# PharmacoForge: pharmacophore generation with diffusion models

**DOI:** 10.3389/fbinf.2025.1628800

**Published:** 2025-09-08

**Authors:** Emma L. Flynn, Riya Shah, Ian Dunn, Rishal Aggarwal, David Ryan Koes

**Affiliations:** 1 Department of Computational and Systems Biology, University of Pittsburgh, Pittsburgh, PA, United States; 2 School of Computer Science, Carnegie Mellon University, Pittsburgh, PA, United States

**Keywords:** structure-based drug discovery, pharmacophore, diffusion models, virtual screening, generative models, molecule generation, computational drug discovery

## Abstract

Structure-based drug design (SBDD) is enhanced by machine learning (ML) to improve both virtual screening and *de novo* design. Despite advances in ML tools for both strategies, screening remains bounded by time and computational cost, while generative models frequently produce invalid and synthetically inaccessible molecules. Screening time can be improved with pharmacophore search, which quickly identifies ligands in a database that match a pharmacophore query. In this work, we introduce PharmacoForge, a diffusion model for generating 3D pharmacophores conditioned on a protein pocket. Generated pharmacophore queries identify ligands that are guaranteed to be valid, commercially available molecules. We evaluate PharmacoForge against automated pharmacophore generation methods using the LIT-PCBA benchmark and ligand generative models through a docking-based evaluation framework. We further assess pharmacophore quality through a retrospective screening of the DUD-E dataset. PharmacoForge surpasses other pharmacophore generation methods in the LIT-PCBA benchmark, and resulting ligands from pharmacophore queries performed similarly to *de novo* generated ligands when docking to DUD-E targets and had lower strain energies compared to *de novo* generated ligands.

## 1 Introduction

Following identification of a disease-causing protein, rational drug discovery aims to design a ligand that binds to the protein target with high specificity and affinity to mitigate disease effects. Structure-based drug design (SBDD) seeks to identify or create a ligand using the molecular structure of a target protein pocket (Anderson, 2003). Computational methods are critical tools in modern SBDD campaigns.

SBDD campaigns are primarily composed of screening-based strategies. Screening-based strategies often involve testing numerous compounds to evaluate their binding to a target protein (Hughes et al., 2011; Blay et al., 2020). Screening is an inherently expensive process regardless of the particular screening method. Direct experimental measurement is exceptionally costly and therefore limited in the size of the chemical space that can be screened. As a result, the use of computational methods to estimate affinity, generally called virtual screening, has become a routine method in drug discovery campaigns over the last several decades Sadybekov and Katritch (2023). Virtual screening methods can evaluate significantly larger chemical spaces than methods based on physical experimentation. Molecular docking, one of the most broadly used virtual screening methods, enables screening of millions or billions of compounds given substantial computing resources, but the screening process remains expensive and time-consuming (Gentile et al., 2020; 2022).

Pharmacophore-based virtual screening is a resource-efficient alternative to molecular docking. Pharmacophore search can be done in sub-linear time, allowing the search of millions of compounds at speeds orders of magnitude faster than traditional virtual screening (Koes and Camacho, 2011; Sunseri and Koes, 2016). A pharmacophore query defines the essential interactions between the ligand and protein where they occur in the binding pocket (Kaserer et al., 2015; Koes, 2015). A molecule matches a pharmacophore query if a valid conformation of the molecule can be positioned such that the essential interactions occur in the correct position. Pharmacophores filter out molecules that do not match the pharmacophore query, which significantly decreases the number of molecules that need to be scored and ranked (Giordano et al., 2022).

The utility of pharmacophore screening results is heavily dependent on the quality of the pharmacophore. Manual pharmacophore design requires identification of potential interaction points in the binding pocket of the receptor either based on the receptor structure or a known reference ligand binding pose; software and automated frameworks have reduced the time and domain-knowledge barriers to improve pharmacophore elucidation processes (Heider et al., 2022). Current pharmacophore design techniques include software implementations, such as Pharmit and Pharmer, that identify interaction points between the protein pocket and a reference ligand and allow user customization of identified centers (Sunseri and Koes, 2016; Koes and Camacho, 2011). Apo2ph4, a framework for pharmacophore elucidation from receptor structure, is proven to perform well in retrospective virtual screening but requires intensive manual checks from a domain expert at each step (Heider et al., 2022). PharmRL, a reinforcement learning method for automated pharmacophore generation, speeds up pharmacophore generation relative to non-automated methods but struggles with generalization and requires training with positive and negative training examples for each protein system (Aggarwal and Koes, 2024). For drug discovery pipelines to fully realize the advantages of pharmacophore screening, user-friendly, automated, and generalizable methods for pharmacophore elucidation are needed.

*De novo* molecule design creates new potential ligands from scratch; *de novo* methods often design ligands based on key structural features of the binding pocket. Techniques include fragment-based drug discovery, which docks smaller building blocks such as a ring structure or amine group into the protein binding pocket then connects them to form a reasonable ligand structure, and traditional methods, which typically involve a combinatorial search (Batool et al., 2019; Durrant et al., 2009).

Applications of generative models to molecule generation have given rise to models capable of predicting *de novo* ligand structures based on the protein pocket (Hoogeboom et al., 2022; Schneuing et al., 2022; Peng et al., 2022; Pinheiro et al., 2024; Ragoza et al., 2022; Dunn and Koes, 2024). Hoogeboom et al. (2022) initially proposed applying equivariant diffusion models to small organic molecules, and various other models, namely, auto-regression-based Pocket2Mol and diffusion-based DiffSBDD, followed to enable conditional generation for a given receptor pocket (Schneuing et al., 2022; Peng et al., 2022). However, several limitations preclude the practical use of these models. *De novo* models that directly condition on 3D structure and assemble individual atoms in a pocket often produce unrealistic or synthetically inaccessible molecules.

We propose circumventing the shortcomings of both virtual screening and *de novo* design methods by leveraging generative modeling to design pharmacophores for a given protein pocket. We introduce PharmacoForge, a diffusion model capable of rapidly generating pharmacophore candidates of any desired size conditioned on a protein pocket of interest. Screening with generated pharmacophores results in matching ligands that are guaranteed to be valid and commercially available. We evaluate generated pharmacophores by both enrichment factor, measuring the ability to identify an enriched subset of active compounds in a database, and docking score of top hits following virtual screening.

## 2 Background

### 2.1 Pharmacophores

**FIGURE 1:**
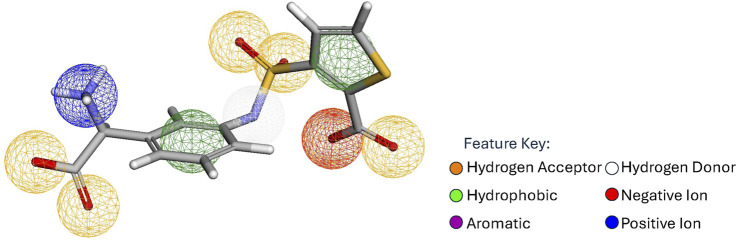
Reference pharmacophore as identified by Pharmit for a ligand binding to AmpC- β -lactamase (PDB 1L2S). Sphere colors correspond to feature type; Blue: Positive Ion, Green: Hydrophobic, Orange: Hydrogen Acceptor, Red: Negative Ion, Purple: Aromatic, White: Hydrogen Donor.

A pharmacophore is a set of points $\left\{V_{f}\right\}$ that represents areas of interactions between a protein and a ligand. Figure 1 shows a reference ligand and its pharmacophore. The areas of interaction are commonly referred to as pharmacophore centers; a pharmacophore’s size is its number of centers. Each pharmacophore center has an associated position $X_{f}\in R^{3}$ and feature type $Z_{f}\in$ {Hydrogen Acceptor, Hydrogen Donor, Hydrophobic, Aromatic, Negative Ion, and Positive Ion}. Collectively, the centers define the spatial and feature constraints that a molecule needs in order to interact with its protein target.

### 2.2 Denoising diffusion probabilistic models

Denoising diffusion probabilistic models (DDPMs) use a Markov process to apply Gaussian random noise to a sample in a noising process and then train a neural network to iteratively denoise the sample (Sohl-Dickstein et al., 2015; Ho et al., 2020). New samples may be drawn from the target distribution by initializing a process from random noise and then iteratively denoising back to a clean sample with the trained model. The noising process can be described by the equation:

$$q\left(x_{t}|x_{0}\right)=N\left(x_{t}|\alpha_{t}x_{0},\sigma_{t}^{2}I\right)$$

Where $x_{0}$ is the data sample, $x_{t}$ is the fully noised sample, $\alpha_{t}$ controls the fraction of original signal maintained, and $\sigma_{t}$ defines the amount of noise added to the data sample at each time step.

### 2.3 Equivariant diffusion models for molecules

Hoogeboom et al. (2022) adapted the Sohl-Dickstein et al. (2015) framework for generative tasks with molecules. Unlike previous applications of diffusion models for image generation, generating molecules introduces the added requirement of E (3)-equivariance. A function is considered equivariant for a group if when the function is applied to both the group, G , and a transformed group, TG , where T represents a transformation, f(TG) is equal to T(f(G)) . For molecules, Euclidean group E (3) transformations (reflection, rotation, and translation) are relevant as molecules retain their identity regardless of any of these transformations; thus models generating molecules must be E (3)-equivariant. Jing et al. (2020) introduced the geometric vector perceptron architecture (GVP), another E (3)-equivariant neural network, as an alternative to standard graph neural networks (GNNs). The standard multi-layer perceptron feed-forward layer in GNNs is replaced by a GVP layer in GVP-GNNs. Unlike GNNs, GVP-GNNs split nodes into scalar and vector channels, adding a directional component which allows more expressive modeling of molecular geometries.

## 3 Related work

### 3.1 Automated pharmacophore generation

Previously proposed automated workflows to generate pharmacophores for a protein pocket, namely Apo2ph4 and PharmRL, apply different computational techniques to create new pharmacophores for virtual screening (Heider et al., 2022; Aggarwal and Koes, 2024). Apo2ph4 primarily relies on fragment docking. The Apo2ph4 workflow identifies a protein pocket based on a provided ligand center of mass or user-specified coordinates then docks 1456 lead-like molecular fragments into the pocket. The docked fragments are filtered to include only those with a docking energy below 2 kcal/mol; a maximum of two poses is kept per successfully docked fragment. Following fragment docking, each selected fragment pose is converted into a pharmacophore; a single pharmacophore is created from the fragment pharmacophores by scoring each center based on proximity to other pharmacophore centers of the same type. Clustering and filtering of proximal centers results in the final pharmacophore for the pocket (Heider et al., 2022).

PharmRL is a reinforcement learning-based pharmacophore generation method which first identifies potential pharmacophore features in a protein pocket by passing a voxelized pocket representation through a CNN. The CNN outputs all possible pharmacophore feature types that an area of the binding pocket may support. The CNN-identified pharmacophore features form the starting point for a deep-Q learning algorithm which iteratively optimizes the features to maximize a reward function and generate a single pharmacophore for the pocket (Aggarwal and Koes, 2024).

### 3.2 Conditional molecular generation

Equivariant diffusion models are capable of generating full ligands unconditionally with no specified properties or conditionally to fit a specific protein pocket. Schneuing et al. (2022) extended the framework of Hoogeboom et al. (2022) to conditional molecule generation with DiffSBDD, an EGNN-based model for molecule generation conditioned on a given protein pocket. Adding protein pocket atoms to the sample graph allows for ligand generation in the context of the protein pocket. Prior to diffusion models’ use in molecular generation, Peng et al. (2022) applied auto-regressive models to the molecule generation task with Pocket2Mol. Pocket2Mol uses an encoder and predictors for molecule coordinates and features.

## 4 Methods

### 4.1 Model implementation details

We trained PharmacoForge using the CrossDocked2020 dataset, which consists of over 18,000 complexes with 22.5 million docked ligand poses (Francoeur et al., 2020). Pharmit is used to identify the interaction pharmacophore centers of a reference protein and ligand complex and build the training dataset of reference pharmacophores and proteins (Sunseri and Koes, 2016). We augment the training dataset by randomly subsampling the ground truth pharmacophore centers provided by Pharmit; a minimum of three and a maximum of eight centers are selected from the pharmacophore. The model is trained with the Adam optimizer at a learning rate of 1e−4 for a total of 80 epochs with a batch size of 24.

### 4.2 Building the pocket-pharmacophore graph

**FIGURE 2:**
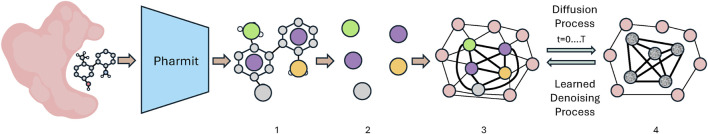
An outline of the protein-pharmacophore graph construction and training process used in PharmacoForge. Proteins and ligands from the CrossDocked dataset are passed through Pharmit to identify the interaction pharmacophore (1). The pharmacophore centers (2) and nearest protein atoms form the protein-pharmacophore graph (3), which then is iteratively noised to train the learned denoising process (4). Created with BioRender.

We represent the protein pocket and pharmacophore as a heterogeneous graph consisting of protein and pharmacophore nodes. A subsampling of the reference pharmacophore centers are added to the graph. We identify the k-nearest neighboring protein atoms to the pharmacophore centers and add those to the final pharmacophore-protein graph. The pharmacophore nodes are fully connected while the protein nodes are connected only to neighboring pharmacophore or protein nodes. Figure 2 illustrates the receptor pocket-pharmacophore graph construction.

Each protein or pharmacophore node has a 3D position represented as $X={\left\{x_{i}\right\}}_{i=1}^{N}\in R^{N\times 3}$ ; pharmacophore nodes have a feature type $F={\left\{f_{i}\right\}}_{i=1}^{N}\in R^{N\times 6}$ while protein nodes have an atom type $A={\left\{a_{i}\right\}}_{i=1}^{N}\in R^{N\times n_{a}}$ , where $n_{a}$ is the number of atom types. Feature types and atom types are both encoded as one-hot vectors.

### 4.3 Diffusion and denoising

We perform an equivariant diffusion process over the heterogeneous graph, noising only the pharmacophore nodes. The protein-pharmacophore graph is embedded into continuous space and then passed through multiple GVP-GNN convolution layers which are used to parameterize the noising process. More details on the GVP-GNN convolutions can be found in the Model Architecture section of Supplementary Material. The variance-preserving noising process follows that of Hoogeboom et al. (2022) (Song et al., 2020; Schneuing et al., 2022):

$$q\left(z_{s}|z_{\mathrm{data}},z_{t}\right)=N\left(z_{t}|\alpha_{t}z_{\mathrm{data}},\sigma_{t}^{2}I\right)$$

Where α is set by a predefined polynomial noise schedule. Both the feature and coordinate vector for each pharmacophore are noised by the same process.

The result of the GVP-GNN convolution layers is passed through a GVP network which predicts the noise added to the sample and obtains the clean coordinates and feature type prediction for each pharmacophore node (Jing et al., 2020). The denoising process also follows that of Hoogeboom et al. (2022) and is learned by optimizing a mean squared error (MSE) loss, where the added noise is predicted and a clean sample is generated by removing the predicted noise.

$$L=\frac{1}{N}\overset{N}{\sum }{\left(\hat{\epsilon }-\epsilon \right)}^{2}$$

where $\hat{\epsilon }$ represents the predicted noise and ϵ represents the true noise. The trained GVP network learns the denoising process and becomes capable of generating clean pharmacophore samples from noise.

### 4.4 Generating pharmacophores

**FIGURE 3:**
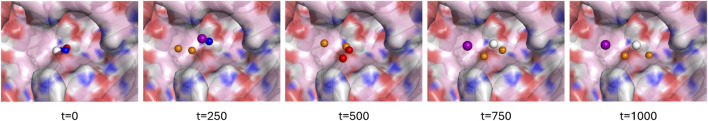
Generating a pharmacophore with four centers for a binding pocket of AmpC- β -lactamase (PDB 1L2S); the pharmacophore is generated over 1000 diffusion time steps (t). Sphere colors correspond to feature type; Blue: Positive Ion, Orange: Hydrogen Acceptor, Red: Negative Ion, Purple: Aromatic, White: Hydrogen Donor.

To generate a new pharmacophore conditioned on a protein pocket, we construct the protein portion of the graph from the pocket atoms; the pocket is identified either through a reference ligand or a list of residues that make up the pocket. We initialize a user-specified number of pharmacophore nodes with random feature vectors and random coordinates near the center of the binding pocket. Using the trained GVP model, we then denoise to get predicted pharmacophore center coordinates and feature types. Figure 3 depicts the pharmacophore generation process from randomly initialized centers to a pharmacophore. The clean pharmacophore is provided to the user as feature types with associated 3D coordinates that can be converted into a pharmacophore query for the protein pocket.

### 4.5 Evaluation metrics

Pharmacophores are difficult to evaluate on inherent value as their utility comes from how accurately and effectively they filter active compounds from a large database. A pharmacophore may correctly identify an area or areas for potential interaction in a protein pocket while still failing to filter enough non-binding ligands to be faster than traditional virtual screening. To assess the accuracy of interactions identified in PharmacoForge during training, we compute pharmacophore validity, which we define as the fraction of centers within a threshold distance of a complementary feature in the binding pocket. The thresholds are based on the interaction type and can be found in the Pharmacophore Validity section of Supplementary Material.

To determine pharmacophore efficacy, we evaluate PharmacoForge-generated pharmacophores based on their ability to find active binders of target proteins and to identify compounds in a pharmacophore search with docking scores competitive with state-of-the-art *de novo* generative models. We assess the generated pharmacophores with both the LIT-PCBA and the Directory of Useful Decoys, Enhanced (DUD-E) benchmarks (Mysinger et al., 2012; Tran-Nguyen et al., 2020). LIT-PCBA contains 15 protein targets with active and decoy compounds for each target. An active compound is a known binder to the target protein; in the LIT-PCBA dataset, all decoys are confirmed inactive compounds for the target (Tran-Nguyen et al., 2020). The DUD-E dataset consists of 102 protein targets and corresponding active and decoy compounds for each target. Decoys included in the DUD-E databases are presumed non-binding compounds (Mysinger et al., 2012).

We query each protein target database with pharmacophores generated for the target and evaluate the results on enrichment factor (EF) and F1 score. EF measures the fraction of active compounds in the total pool of ligands identified relative to the fraction of active compounds present in the queried database.

$$EF=\frac{\text{Fraction of actives in query results}}{\text{Fraction of actives in database}}$$

An EF score of one indicates the pharmacophore query result is equal to selecting ligands from the database at random; above a one indicates the query result contains an enriched subset of actives.

An F1 score is the geometric mean of precision and recall and considers true positives (actives identified by the query), false positives (decoys in the query result), and false negatives (actives present in the database not included in the query result) (Aggarwal and Koes, 2024). Precision and recall are used to calculate the F1 score as shown below:

$$\text{Precision}=\frac{\text{TP}}{\text{TP}+\text{FP}}$$

$$\text{Recall}=\frac{\text{TP}}{\text{TP}+\text{FN}}$$

$$F1=\frac{2*\text{Precision}*\text{Recall}}{\text{Precision}+\text{Recall}}$$

where TP (true positive) is an active in the database that appears in the query result, FP (false positive) is a decoy that appears in the query result, and FN (false negative) is an active in the database that does not appear in the query result.

We also assess pharmacophores by their performance in a pharmacophore search. We seek to identify ligands with a high binding affinity for the DUD-E target proteins by querying a large chemical database of potential ligands. To evaluate the ligands, we perform minimization and docking using GNINA (McNutt et al., 2021), a fork of the AutoDock Vina (Trott and Olson, 2010) docking software that uses a convolutional neural network to score protein-ligand interactions. Minimization finds the local optimal pose for a ligand bound to a protein, while docking searches for the global optimal bound pose.

A ligand’s binding affinity for the protein is gauged by multiple scores provided by GNINA: Vina score, CNN affinity, and CNN VS score. The Vina score is the predicted affinity of the ligand for the protein in kJ/mol. CNN affinity is the affinity score predicted by GNINA using a CNN. The CNN VS score is the CNN affinity multiplied by the CNN score, which predicts the pose probability, and represents the affinity of the ligand in the pose as well as the likelihood that pose would occur (Sunseri and Koes, 2021). We compare our pharmacophore query results to *de novo* generated ligands across all GNINA scores after selecting the top ligands based on Vina affinity score.

## 5 Results

### 5.1 Comparison to other pharmacophore generation methods

We compare PharmacoForge to two automated pharmacophore generation methods, PharmRL and Apo2ph4, using the LIT-PCBA baseline. Apo2ph4 reported screening results for pharmacophores generated for the 15 LIT-PCBA targets, selecting 20 PDBs of the full dataset. PharmRL created pharmacophores and performed screening for the same target PDBs. Because screening with Apo2ph4 pharmacophores was originally conducted with proprietary software, Aggarwal and Koes (2024) used the pharmacophores provided by Apo2ph4 and screened instead with Pharmit.

**FIGURE 4:**
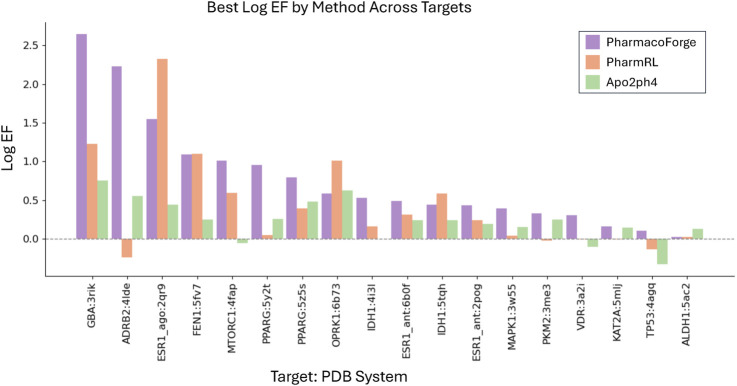
Log EF results for the LIT-PCBA benchmark for PharmacoForge, PharmRL, and Apo2ph4. Each bar represents the highest Log EF achieved by a query based on each method for each target in the benchmark.

**FIGURE 5:**
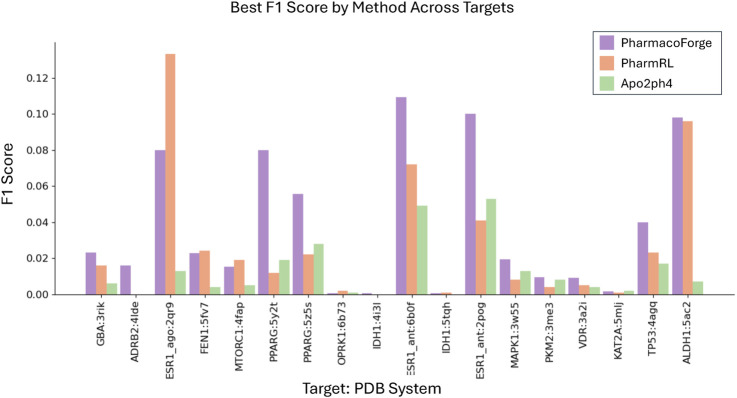
F1 results for the LIT-PCBA benchmark for PharmacoForge, PharmRL, and Apo2ph4. Each bar represents the highest F1 achieved by a query based on each method for each target in the benchmark.

To compare with Apo2ph4 and PharmRL, we generated five pharmacophores of each size 3–8 centers for a total of 30 pharmacophores. We then screened with Pharmit using receptor exclusion to be consistent with the other two methods; receptor exclusion ensures that identified ligands in the pharmacophore search do not overlap with the protein receptor. We compared the results for each method as reported in PharmRL, selecting the best performing pharmacophore based on highest F1 score and breaking any ties with the greatest EF score; similarly for PharmacoForge, the pharmacophore for comparison was selected based on top F1 score with EF score deciding any ties. We report the EF and F1 scores of all methods in Figures 4, 5. PharmacoForge-generated pharmacophores perform best in screening based on F1 scores for 12 out of 18 targets while PharmRL has the top score for five targets and Apo2ph4 for one; when comparing by EF, PharmacoForge achieves the best result for 13 out of 18, PharmRL for four, and Apo2ph4 for one.

PharmacoForge creates at least one pharmacophore with an EF above one for all targets in LIT-PCBA, which is not true of either PharmRL or Apo2ph4; furthermore, PharmacoForge queries achieve an F1 score above zero at least once for all targets. The performance on both metrics demonstrates that PharmacoForge generates informative pharmacophores that perform comparatively well in a pharmacophore search.

### 5.2 Identifying active compounds with generated pharmacophores

For this benchmark we sought to identify an enriched subset of active compounds from the DUD-E target databases using a generated pharmacophore query. We first generate pharmacophores with 3–8 centers for each target conditioned on the reference receptor PDB provided by DUD-E; we sample five pharmacophores of each size for a total of 30 pharmacophores per target. We then query a database of DUD-E ligands for each target containing both actives and decoys and calculate the EF and F1 score of each query result. We perform the database queries using Pharmit, which identifies ligands in the database that match the pharmacophore centers. Pharmit generates 25 conformers per ligand in the database to compare the pharmacophore against, but we limit the number of conformers returned in the query results to one per molecule. For a molecule to match a pharmacophore query, the conformer pose must contain interaction features that align to within 1 Å RMSD of all pharmacophore centers. We compare the generated pharmacophores’ performance to a reference set of pharmacophores constructed by randomly subsampling the reference pharmacophore for each target. The reference pharmacophore centers capture ground truth interaction features between the ligand and protein and offer an approximation of how well a pharmacophore may perform as a query; however, not all reference ligands provided by DUD-E are included as actives in the target database. The reference ligand is only included as an active for 19 out of 102 targets, so a reference pharmacophore may not necessarily match an active compound despite containing ground truth interactions that would allow a ligand to bind. The reference pharmacophore set represents an informative basis for comparison as it contains accurate interaction points for the protein that have the potential to match multiple active scaffolds but is still limited in the diversity of ligand scaffolds that match. A reference pharmacophore is found by Pharmit based on the reference protein and ligand PDB and SDF files provided by DUD-E. To obtain a reference set of 30 pharmacophores, we created five pharmacophores of each size 3–8 centers by randomly selecting the desired number of centers. We again queried the DUD-E target databases with the reference pharmacophores using Pharmit and computed the EF and F1 scores of each query result.

**FIGURE 6:**
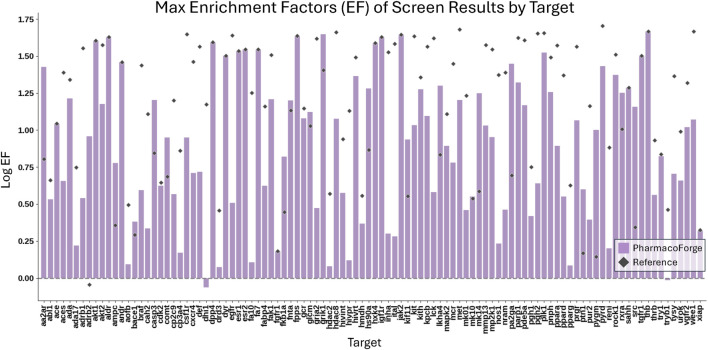
Comparison of the maximum EF scores for each target of 30 pharmacophores generated by PharmacoForge and 30 pharmacophores subsampled from the reference ligand provided by DUD-E.

**FIGURE 7:**
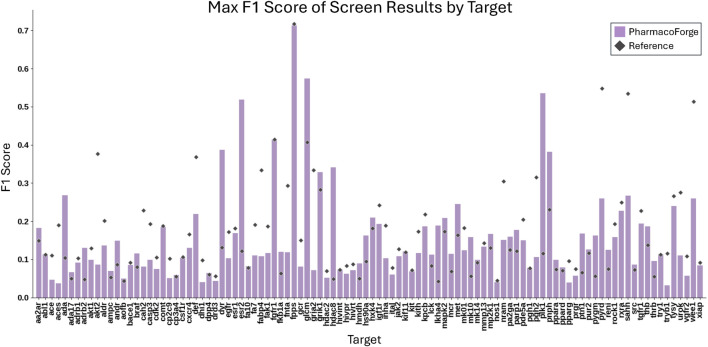
Comparison of the maximum F1 scores for each target of 30 pharmacophores generated by PharmacoForge and 30 pharmacophores subsampled from the reference ligand provided by DUD-E.

The full resulting EF and F1 scores of both generated and reference pharmacophores are shown in Additional DUD-E Screening Benchmark Results section of Supplementary Material. Pharmacophore queries that returned no results (undefined EF) are excluded from the EF analysis. 1584 reference and 1229 generated pharmacophore queries returned no results, representing 52% and 40% of total queries, respectively. Reference pharmacophores failed to find actives in 63% of queries; generated pharmacophore queries returned no actives in 58% of queries. We compare the average and maximum EF and F1 scores of reference and generated pharmacophores across all pharmacophore queries that returned this result is shown in Figures 6, 7.

Table 1 reports the number of targets for which the generated or reference pharmacophores achieved a higher mean or maximum EF or F1 score. The generated pharmacophores achieved an average EF greater than that of the reference pharmacophores on 18 out of 102 targets and had a maximum EF greater than or equal to the reference maximum EF on 37 out of 102 targets. Outperforming the reference pharmacophores for some targets demonstrates that the generated pharmacophores are informative and capable of finding enriched subsets of active compounds for a target. The generated pharmacophores often outperform the reference pharmacophores on average F1 score and perform similarly on maximum F1 score. The improvement in F1 scores for generated pharmacophores over reference results from a higher average recall on 95 out of 102 targets.

**TABLE 1:** Number of targets for which Generated or Reference pharmacophores have the best result for each metric; Equal indicates when the Generated and Reference pharmacophores had the same value for the metric on a target.

	Avg EF	Max EF	Avg F1	Max F1
Generated	18	20	71	46
Reference	84	65	31	56
Equal	0	17	0	0

### 5.3 Pharmacophore-matching ligand comparison with *de novo* ligand methods

Calculating the EF and F1 scores of pharmacophore query results provides useful metrics of how well the pharmacophore matches known active ligands, but a pharmacophore may still identify useful interactions while not matching previously identified congeneric series of actives. To further evaluate the quality of the compounds selected through our generated pharmacophores, we use docking scores as a proxy for binding affinity. We evaluated the results of a pharmacophore query by minimizing and docking the result ligands to the target protein and comparing the predicted affinity scores. A more negative affinity value suggests that the ligand is an active binder of the target. We minimized and docked the filtered query results to their respective target proteins using GNINA (McNutt et al., 2021).

For this analysis, we sought to identify hit molecules for the DUD-E targets and screened the CHEMBL database, which we downsampled from two million compounds to 200,000 compounds. Using our previously generated 30 pharmacophores for each target, we queried CHEMBL and selected pharmacophores with query results of 2000 or fewer molecules; this cutoff represents 1% of the queried database size and was used to eliminate pharmacophores lacking specificity of results. This amounted to 1175 pharmacophore queries remaining. Each returned hit is minimized with GNINA to find the local optimal solution most aligned with the pharmacophore. We also docked the query results with GNINA for an approximation of global optimal pose of the identified compound and direct comparison with our random baseline. We then identified the top 100 ligands based on Vina affinity score for each target to compare against other methods.

#### 5.3.1 Baselines

We compared PharmacoForge-identified CHEMBL compounds with ligands generated by two *de novo* ligand generative models, Pocket2Mol and DiffSBDD. These models were chosen based on their high performance relative to other *de novo* ligand generative models and availability of a trained model (Schneuing et al., 2022; Peng et al., 2022). As a further baseline, we also randomly selected 10,000 molecules from CHEMBL and docked those to each DUD-E target for comparison.

For each generative model, we used the default settings of each model to sample 1000 ligands for each target; for some targets, DiffSBDD and Pocket2Mol were unable to generate 1000 unique ligands, but at least 850 ligands were generated for all targets. We then minimized and docked each ligand to its corresponding receptor. We again selected the best 100 ligands based on Vina affinity score for each target and compared to the affinity scores for our pharmacophore query result ligands.

#### 5.3.2 Ligand strain energies

**FIGURE 8:**
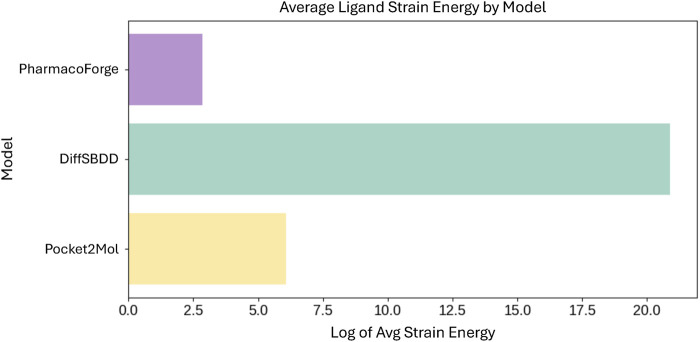
Comparison of average strain energies for ligands in minimized poses. Average strain energy shown on log scale.

Further analysis and visualization of all ligand results revealed that *de novo* generated ligands were sometimes wedged in the target protein pocket in highly strained poses. To evaluate the strain of both pharmacophore queried ligands and *de novo* generated ligands, we calculated the total energy of each molecule before and after geometry optimization using the Universal Force Field (UFF) as implemented in RDKit (Landrum et al., 2024). The strain is quantified as the energy difference between the unoptimized and optimized structures. Pharmacophore queried ligands exhibited a median strain value of 0.05 kcal/mol, with only small energy reductions observed upon optimization. In comparison, DiffSBDD and Pocket2Mol had median strain energies of 295.7 kcal/mol and 351.5 kcal/mol, respectively. The difference in average strain energies of each set of ligands is visualized in Figure 8. The orders of magnitude difference in strain energies between methods indicates that PharmacoForge retrieves commercially available molecules in realistic conformations, improving on an existing problem with *de novo* 3D molecule generative methods.

#### 5.3.3 Ligand minimization with GNINA

**FIGURE 9:**
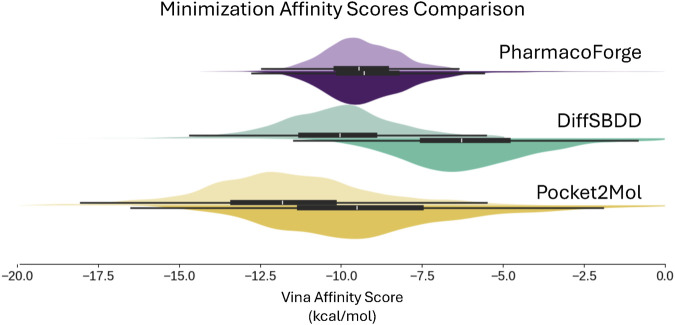
Distribution of Vina docking scores from minimization, which evaluates generated poses at a locally optimum configuration close to the generated pose. Results shown for the top 100 ligands for each DUD-E target. Original pose Vina scores on top half of each violin plot with de-strained Vina scores on the bottom half.

The results of minimizing the de-strained molecules are shown in Figure 9 and reported in Table 2. Ligands identified with pharmacophore search from generated pharmacophores have similar predicted affinity for the target proteins as generated ligands. The strained predicted affinities for the best 100 ligands are similar between PharmacoForge and DiffSBDD results; Pocket2Mol compounds have the best average affinity for the targets. After de-straining, compounds from both DiffSBDD and Pocket2Mol see decreased affinity for the targets as indicated by a larger Vina score, with Vina scores of DiffSBDD ligands increasing by 5.1 kcal/mol and Pocket2Mol by 3.2 kcal/mol. The Vina score for PharmacoForge increases by just 0.37 kcal/mol by comparison, and the de-strained results have the highest affinity for the targets. PharmacoForge is less impacted by the issue of highly strained ligands seen with purely generative models. PharmacoForge also has a narrower distribution compared to the predicted affinity range of both DiffSBDD and Pocket2Mol, with a standard deviation for de-strained predicted affinity of 2.13 compared to 5.15 and 2.56, respectively; PharmacoForge achieves more consistent results across targets.

**TABLE 2:** Minimization mean scores comparison between original and de-strained poses with standard error.

	Model	Affinity (Vina) ↓	CNN affinity ↑	CNN VS score ↑
Original Poses	Pocket2Mol	−11.69±0.03	7.12±0.01	3.92±0.03
	DiffSBDD	−10.06±0.02	7.27±0.01	4.14±0.02
	**PharmacoForge**	−9.38±0.01	7.05±0.01	2.95±0.02
Destrain Poses	Pocket2Mol	−8.45±0.05	7.35±0.01	2.06±0.02
	DiffSBDD	−4.94±0.05	6.34±0.01	2.19±0.02
	**PharmacoForge**	−9.01±0.02	6.99±0.01	2.82±0.02

Best score for each column is listed in bold.

#### 5.3.4 Ligand docking with GNINA

**FIGURE 10:**
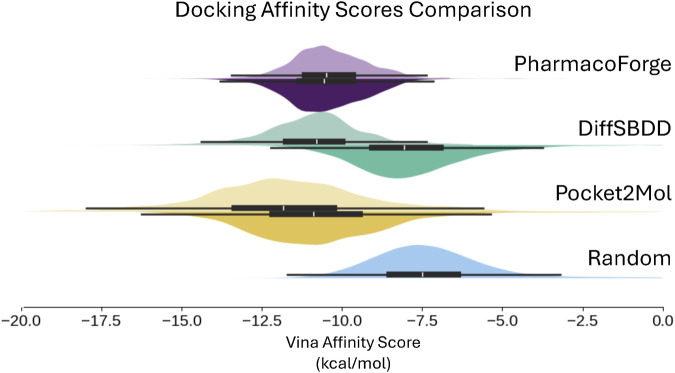
Distribution of predicted binding affinity from docking, which does not use the initial generated pose and so results can be meaningfully compared to a random sample of compounds. Results shown for the top 100 ligands for each target. Original pose Vina scores on top half of each violin plot with de-strained Vina scores on the bottom half. Random baseline includes only original scores.

The docking result shown in Figure 10 and Table 3 includes a random baseline of CHEMBL compounds for comparison; the randomly selected compounds were kept in their original pose and not de-strained. All methods surpass the random baseline for both original and de-strained poses by at least −5.5 kcal/mol. As also seen in minimization results, PharmacoForge and DiffSBDD perform comparably while Pocket2Mol ligands have the greatest affinity for the target proteins. De-straining of the ligands results in an increased Vina score, indicating a decrease in predicted affinity for DiffSBDD and Pocket2Mol, while de-straining PharmacoForge found ligands leads to a slight improvement in predicted affinity. The Vina score increases for DiffSBDD and Pocket2Mol by 3.2 kcal/mol and 1.1 kcal/mol, respectively. The ligands identified through pharmacophore search have high predicted affinity for their targets while maintaining a natural pose, and de-straining of the pose does not lead to a loss of affinity for the target as seen in generated ligands.

**TABLE 3:** Docking mean scores comparison between original and de-strained poses with standard error.

	Model	Affinity (Vina) ↓	CNN affinity ↑	CNN VS score ↑
Original Poses	Random	−5.08±0.01	6.70±0.00	3.63±0.0
	Pocket2Mol	−11.73±0.03	8.01±0.01	6.66±0.02
	DiffSBDD	−10.83±0.02	8.55±0.02	6.66±0.01
	**PharmacoForge**	−10.39±0.01	8.40±0.12	8.30±0.09
Destrain Poses	Pocket2Mol	−10.59±0.03	7.71±0.02	3.68±0.04
	DiffSBDD	−7.65±0.03	6.80±0.03	4.06±0.03
	**PharmacoForge**	−10.49±0.02	7.73±0.02	4.98±0.04

Best score for each column is listed in bold.

## 6 Conclusion

In this work, we presented PharmacoForge, which generates novel pharmacophores conditioned on a protein pocket. Generating pharmacophores leverages the power of generative modeling to create a structural description of the desired molecules that can be used to rapidly screen libraries of valid, commercially available, synthetically accessible molecules.

Our pharmacophore screening results surpass existing automated pharmacophore generation methods and are comparable with existing methods for *de novo* ligand generation without suffering from high strain. Further additions to predict directionality for relevant pharmacophore features as well as learned model-determined pharmacophore size may improve the screening performance.

Automated pharmacophore elucidation eliminates barriers to further adoption of pharmacophore screening in drug discovery campaigns to allow for accelerated screening of large chemical databases. The interpretability of pharmacophores enables human-in-the-loop discovery where experts work with generative models to ultimately uncover commercially available leads for drug discovery. Recent work in generative models for *de novo* ligand design create new ligands based on a pharmacophore, which has led to improvements in validity and target affinity for generated ligands (Ziv et al., 2025; Zhu et al., 2023; Wang and Rajapakse, 2024; Wang et al., 2022; Imrie et al., 2021). Accurately identifying key ligand-protein interactions in the binding pocket allows for better informed ligand generation but pharmacophore-based ligand generative models require a high-quality pharmacophore to be effective. Automating pharmacophore generation can directly complement these efforts by enabling a fully-automated ligand generation pipeline that produces higher quality ligands than current ligand generative models. Automated pharmacophore generation has immediate value as an aid to existing virtual screening pipelines and holds promise as an important step in future ligand generative models.

PharmacoForge is available for use as a Google Colaboratory notebook here, and the full model implementation and open source training code are available at https://github.com/eflynn8/pharmacophore-diffusion.

## Data Availability

The original contributions presented in the study are included in the article/Supplementary Material, further inquiries can be directed to the corresponding author.
