# Supplementary material for: PharmacoForge: pharmacophore generation with diffusion models
Source: Front Bioinform. 2025 Sep 8;5:1628800. doi: 10.3389/fbinf.2025.1628800 (PMC12451294; doi:10.3389/fbinf.2025.1628800)
Supplement: Supplementary file 1 [file DataSheet1.pdf]

Supplementary Material

1 MODEL ARCHITECTURE

The neural network takes as input a noised protein-pharmacophore graph, with noise added only to coordinates and feature types of pharmacophore nodes, and is tasked with predicting the noise added to the graph. For both the pharmacophore coordinates and feature types, we train the model with an MSE loss.

The protein and pharmacophore, along with the diffusion time step, are first embedded into a continuous space, which is then taken as input through several GVP-GNN convolution layers; the output of the GVP-GNN layers is then passed through the noise prediction block to obtain the noise predictions.

1.1 GVP-GNN convolution layer

A GVP-GNN convolution layer accepts and returns tuples of scalar and vector features. It does this by generating scalar and vector messages $m^{(s)}_{i\to j}$ and $m^{(v)}_{i\to j}$. The message updates are given by

$$m^{(s)}_{i\to j}, m^{(v)}_{i\to j} = g\left([s_i; \mathrm{rbf}(d_{ij})], \left[v_i; \frac{x_i - x_j}{d_{ij}}\right]\right) \tag{S1}$$

where ; represents concatenation, g is the chaining of 3 GVP layers, and rbf is a radial basis function embedding of the distance between the two nodes (d_{ij}).

The nodes are updated according to:

$$s_i^{(l+1)}, v_i^{(l+1)} = \left([s_i^{(l)}, v_i^{(l)}] + \left(g_u\left(\frac{1}{|\mathcal{N}(i)|}\sum_{j\in\mathcal{N}(i)}\left[m^{(s)}_{j\to i}, m^{(v)}_{j\to i}\right]\right)\right)\right) \tag{S2}$$

where g_u is a chaining of two GVP layers.

1.2 Noise prediction block

The output of the GVP convolutions results in scalar and vector features for each node. These are sent through four GVP layers, where the first three provide the same number of scalar and vector features as the input while the final layer return only one vector feature. This last vector is taken as the prediction for noise added to the coordinates; the clean coordinates are obtained by subtracting the predicted noise from the noised coordinates. The output scalar features from the GVP layers are sent through an MLP to obtain the prediction for feature types.

1.3 Dataset Processing

We train the diffusion model on the CrossDocked dataset (Francoeur et al., 2020). We utilize the cross validation splits provided with the CrossDocked dataset. To prune the size of our dataset, we work with only the binding pockets of each data point. To specify the binding pocket, we extract all residues within 8 Å of any ligand atom and remove all hydrogen atoms. The protein atoms are then featurized by the element type and their 3D positions. The ground truth pharmacophores are obtained by running Pharmit (Sunseri and Koes, 2016) on the complexes present in the dataset. The exact command is as follows:

```
pharmit pharma -receptor {rec_path} -in {lig_path} -out {phfile}
```

1.4 Training Details

The model is trained with the Adam optimizer at a learning rate of 1e-4 for a total of 80 epochs at a batch size of 24. The training data is augmented by randomly subsampling ground truth pharmacophore centers. We ensure that the minimum size of the pharmacophore is three and the maximum size is eight when augmenting the dataset.

1.5 Model Hyperparameters

All the GVP convolutions use 128 scalar features and 16 vector features. The edges in the graph are assigned based on the distance constraints in Table S1.

Table S1. Edge Assignment Methods, P are protein nodes, and F are pharmacophore nodes

Edge Type	Distance Threshold	Neighbor Threshold
$P \rightarrow P$	8	None
$F \rightarrow P$	8	5
$F \rightarrow F$	100	None

A neighbor threshold ensures that the node does not have more than that number of edges of that particular edge type, essentially making it a distance constrained K-NN graph. The high distance threshold for $F \rightarrow F$ (pharmacophore to pharmacophore) ensures that pharmacophores are fully connected.

2 PHARMACOPHORE VALIDITY

During training, we assess pharmacophore validity by computing the complementarity of generated pharmacophore features to interaction features present in the protein pocket. The protein node interaction type is determined by neighboring pharmacophore nodes in the protein-pharmacophore reference graph. For each pharmacophore center, we compute the distance between the pharmacophore node and protein nodes with a complementary feature and determine if at least protein node is within the distance threshold required for that interaction to occur. The distance thresholds and complementary interactions for each pharmacophore feature type are described in Table S2. The final computed validity for our trained model was 0.89 for pharmacophore generated for the full training set.

Table S2. Distance thresholds and complementary interaction types for pharmacophore feature complementarity based on feature type

Feature Type	Distance Threshold (Å)	Complementary Interactions
Aromatic	7	Aromatic, Positive Ion
Hydrogen Acceptor	5	Hydrogen Donor
Hydrogen Donor	4	Hydrogen Acceptor
Hydrophobic	4	Hydrophobic
Negative Ion	4	Positive Ion
Positive Ion	4	Negative Ion, Aromatic

3 PHARMACOPHORE SIMILARITY

To assess the diversity of pharmacophore samples, we constructed a similarity metric to calculate a similarity score between any two pharmacophores. Given a list of points for two pharmacophores containing 3D coordinates and feature type, we compute the distance between pharmacophore centers of the same type and add those below a threshold of 2Å to a list of potential matches. This threshold was selected because the tolerance for a pharmacophore center is 1Å, so a ligand feature could only be matched to either center if they are no more than 2Å apart.

We then apply the Gale-Shapley algorithm to the lists of potential matches to identify a single best match for each pharmacophore center where possible (Gale and Shapley, 1962). A pharmacophore center can have no more than a single match on the other pharmacophore; when pharmacophores are of different sizes, the Gale-Shapley algorithm is applied to be optimal for the smaller pharmacophore, meaning all the smaller pharmacophore's centers will be matched with the best matches possible. Once the pharmacophore centers have been matched, we compute the Tanimoto similarity, or Jaccard index, based on the total number of centers between the pharmacophores.

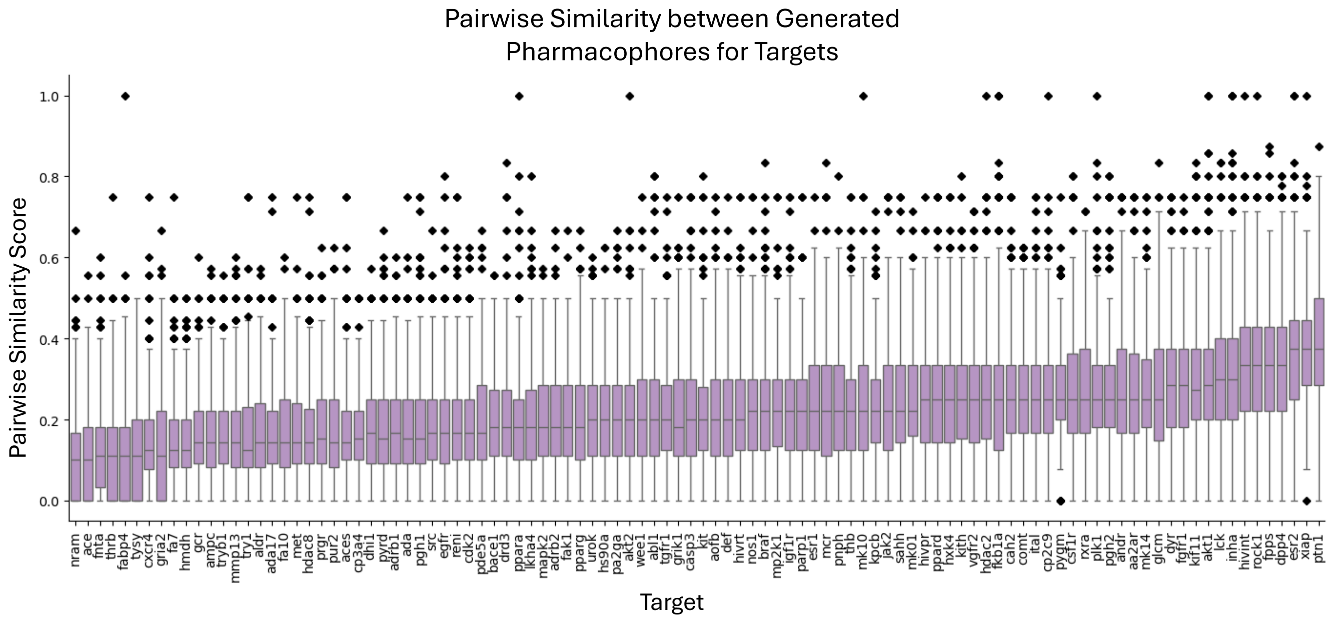

Figure S1. Distributions of similarity scores for pairwise similarity between generated pharmacophores for the same target; targets are ordered based on mean similarity score.

We calculate the pairwise similarity between all pharmacophores generated for the same pocket and also compare each generated pharmacophore to its corresponding reference pharmacophore. The results of comparing among generated pharmacophores is shown in Figure S1 and the comparison between generated and reference is shown in Figure S2. The average pairwise similarity of generated pharmacophores is below 0.4 for all targets. The average mean similarity across targets is 0.22 when comparing within generated pharmacophores and falls to 0.13 when comparing between generated and reference pharmacophores. The average maximum similarity between pharmacophores for a target is 0.75; the average maximum similarity between generated and reference pharmacophores is 0.60. These values indicate that PharmacoForge is

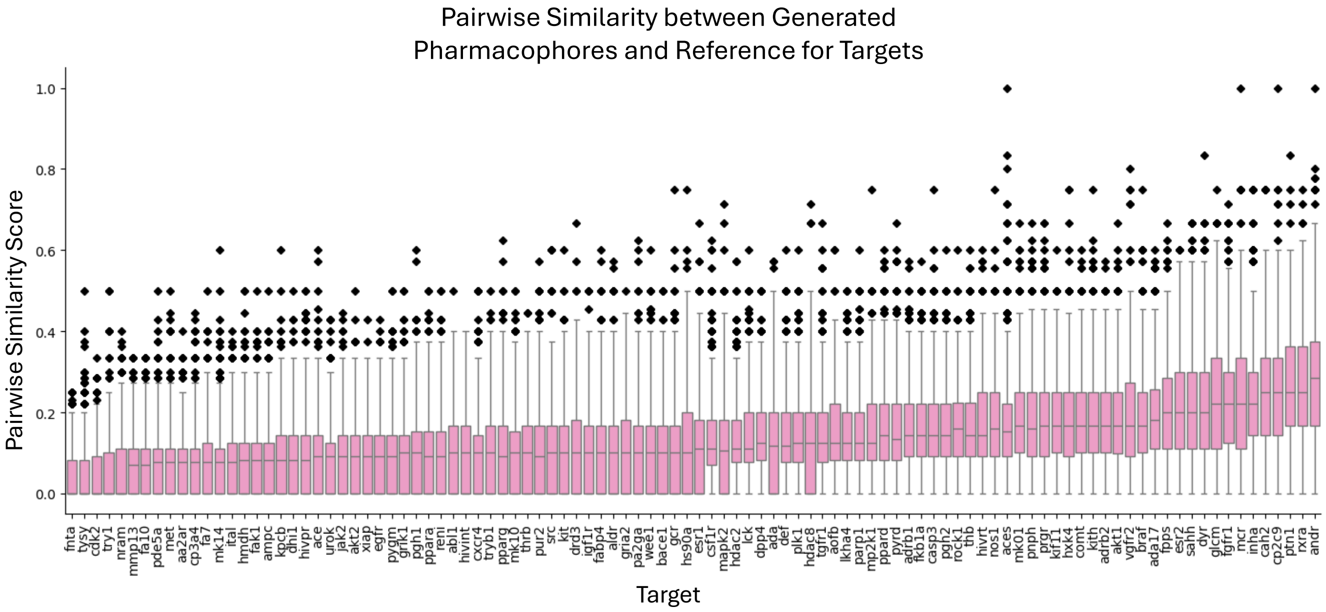

Figure S2. Distributions of similarity scores for pairwise similarity between generated and reference pharmacophores for the same target; targets are ordered based on mean similarity score.

capable of generating a diversity of pharmacophores for each target that are not simply replicas of the reference pharmacophore.

4 ADDITIONAL DUD-E SCREENING BENCHMARK RESULTS

The full results for EF and F1 scores on the DUD-E benchmark can be seen in Figures S3 and S4. Each point represents the average metric of the five pharmacophores of that size for that target. The plots are ordered by decreasing metric in the reference pharmacophores. We see that generated pharmacophores often approach performance of reference pharmacophores of similar size in both metrics. Pharmacophores of larger size tend to result in queries with higher EF for both generated and reference pharmacophores. This is not true for F1 scores, where smaller pharmacophores that capture a larger number of total actives present tend to perform better.

In addition to comparing the average and maximum query metrics across targets, we also computed metrics for the full results from all queries. For each target, we appended the results of all 30 queries, removed duplicate molecules, then calculated the EF and F1 score; we also report the recall. From these results, we can gauge what fraction of total actives present in the database the generated pharmacophores are able to capture. No target reaches an EF above 1 based on the full result. The F1 scores are more consistent with average F1 scores from each query; the average of all F1 queries individually is 0.024 while the average F1 score of the full query results combined is 0.037. We further examine the recall of the union query results which represents the fraction of total actives identified by the queries. The queries achieve an average recall of 0.55, with a minimum of 0.13 and a maximum recall of 0.88. These results are shown in Figures S7, S8, and S9.

We also compare generated pharmacophore performance between pharmacophores generated for DUD-E targets present in the CrossDocked dataset and those not present. 77 out of 102 targets are present

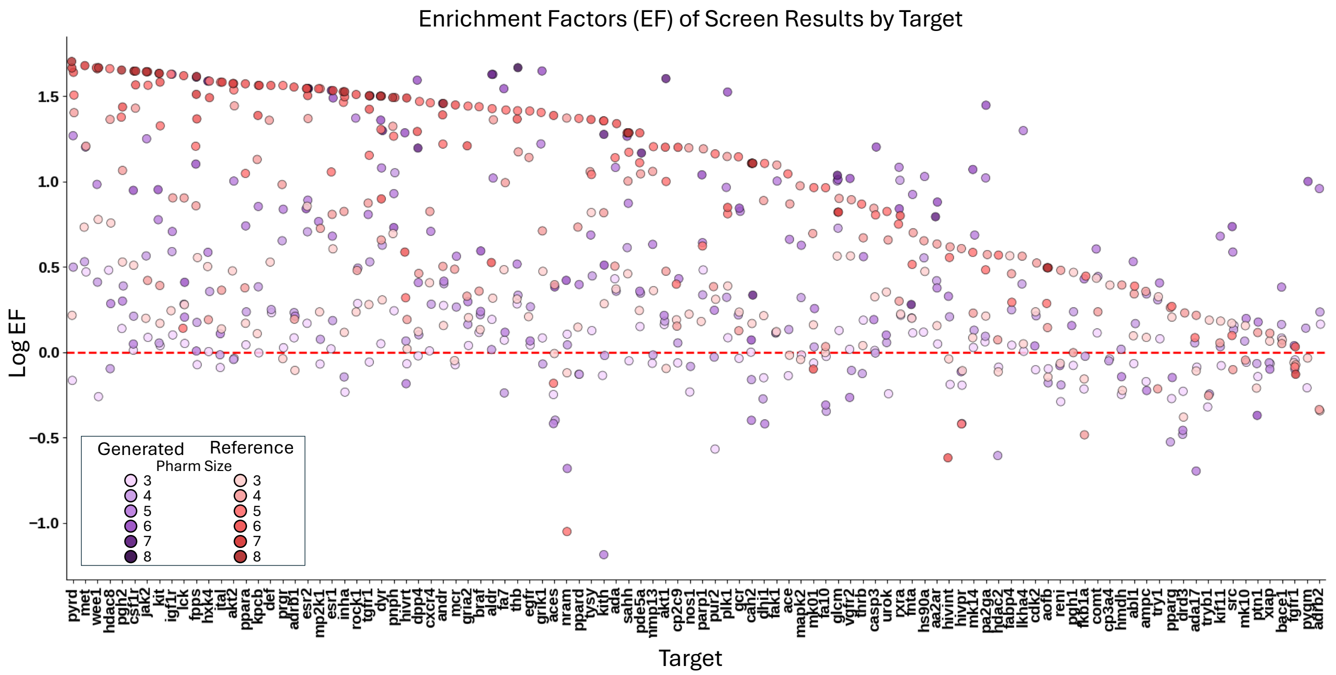

Figure S3. Full EF results of pharmacophore queries for all generated pharmacophores. Excludes queries that returned zero results.

in CrossDocked, but the distribution of average EF is relatively consistent across targets in and not in CrossDocked.

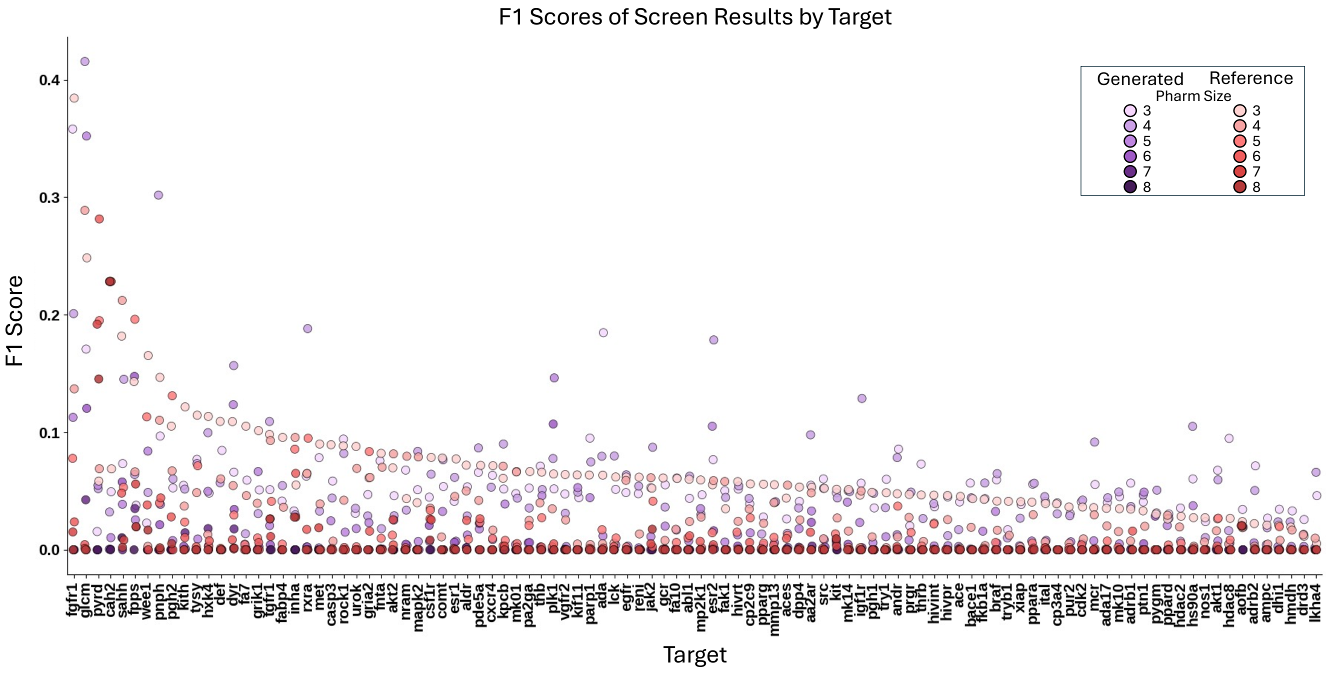

Figure S4. Full F1 results of pharmacophore queries for all generated pharmacophores. Excludes queries that returned zero results.

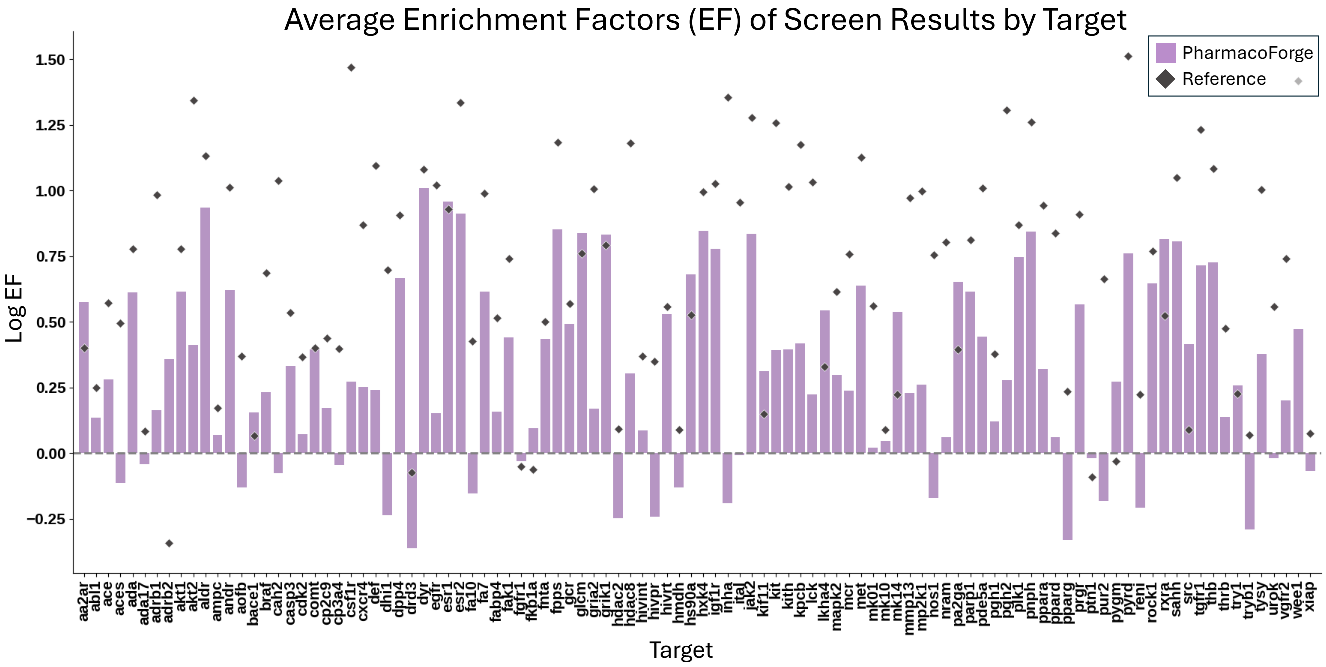

Figure S5. Average EF of all 30 pharmacophores for each target.

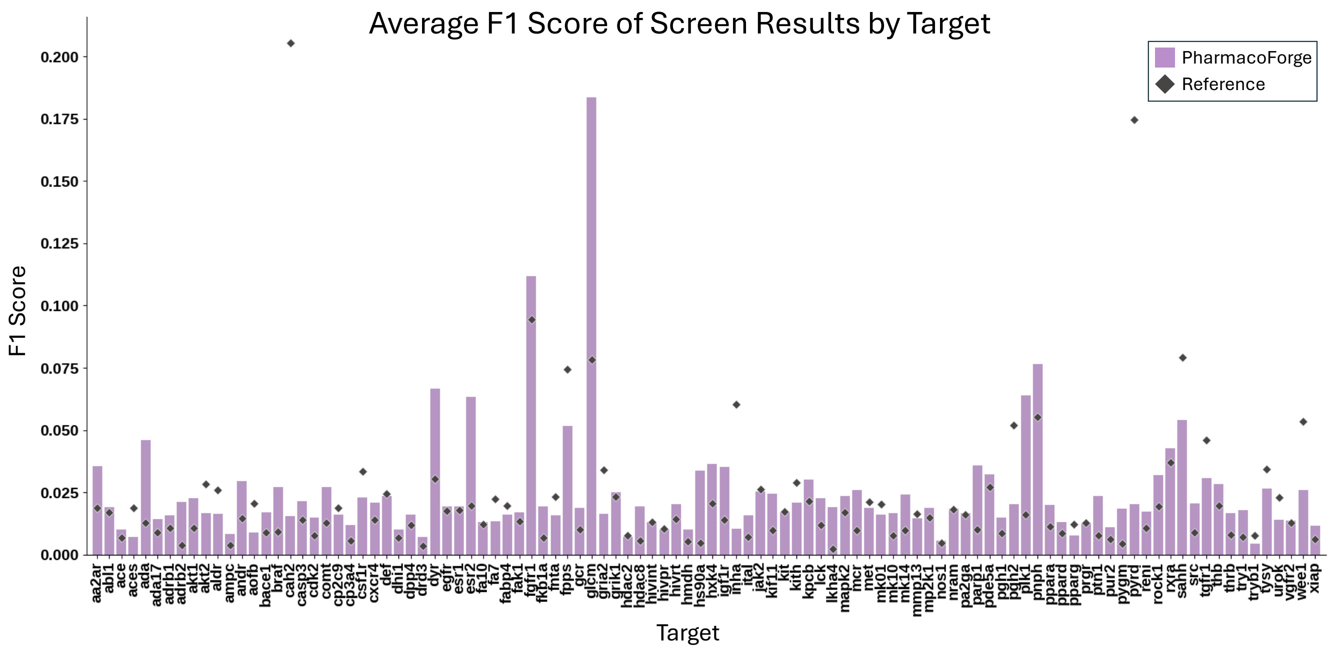

Figure S6. Average F1 scores of all 30 pharmacophores for each target.

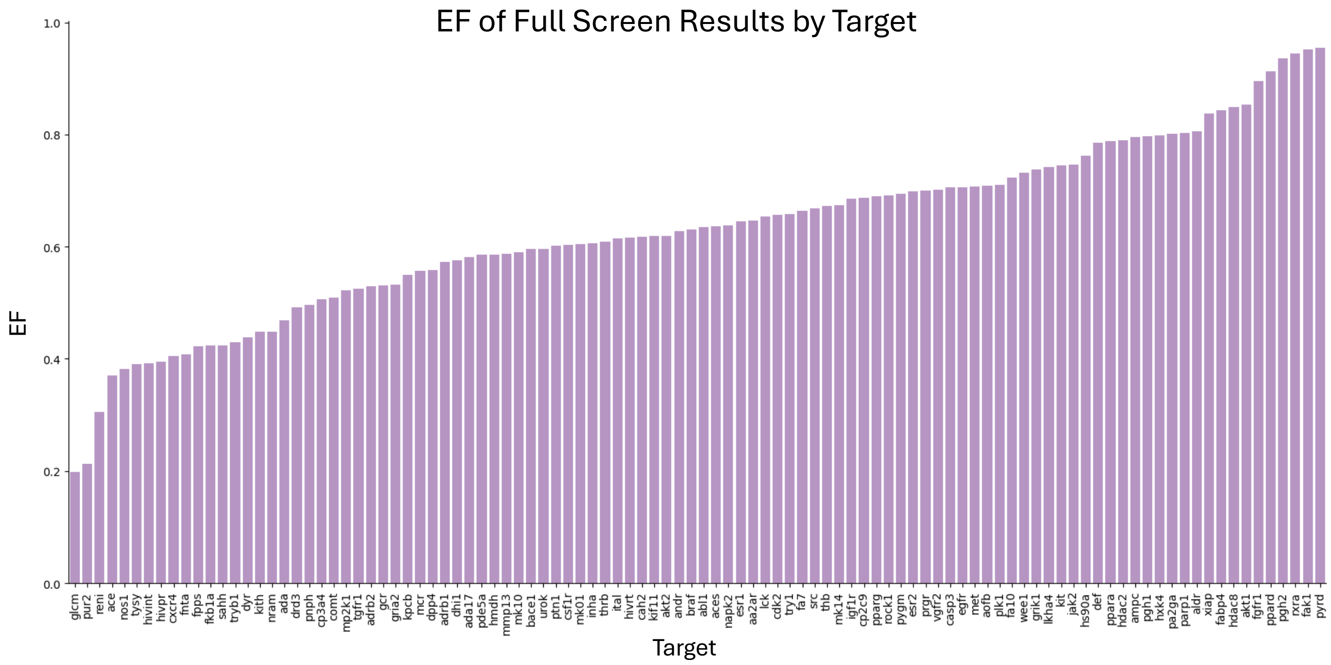

Figure S7. EF values for full results across all queries for a target. Results were combined and then duplicates were removed.

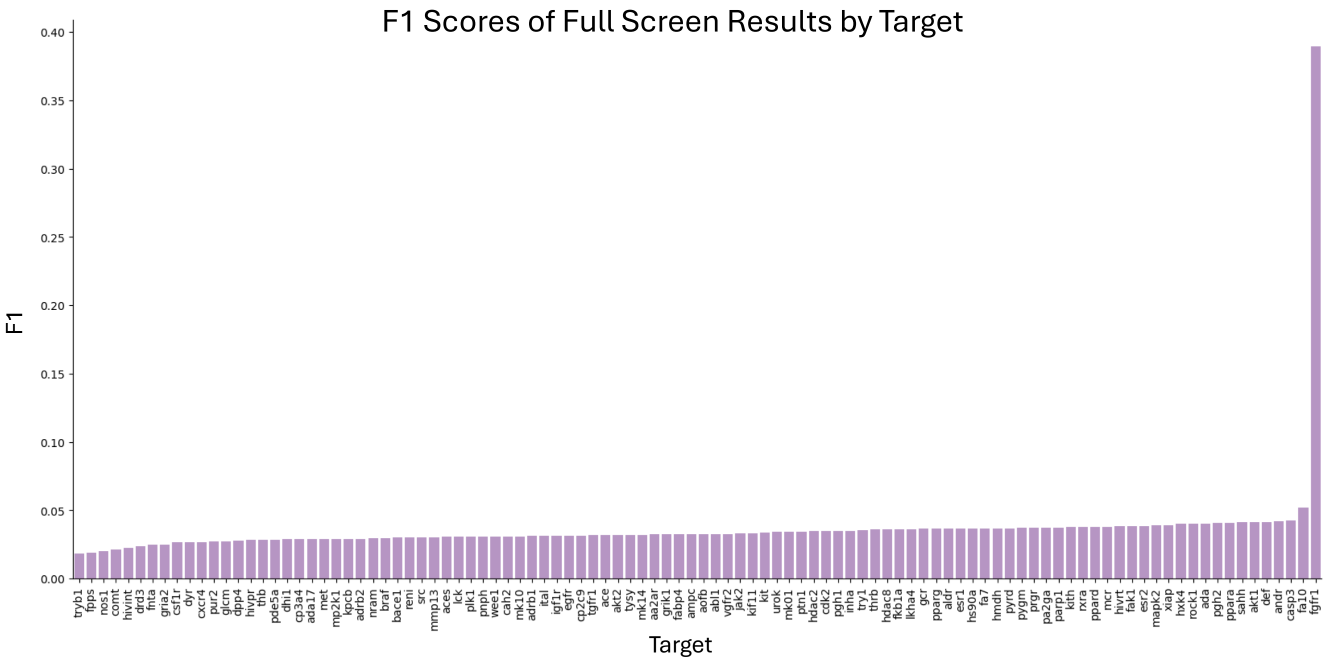

Figure S8. F1 scores for full results across all queries for a target. Results were combined and then duplicates were removed.

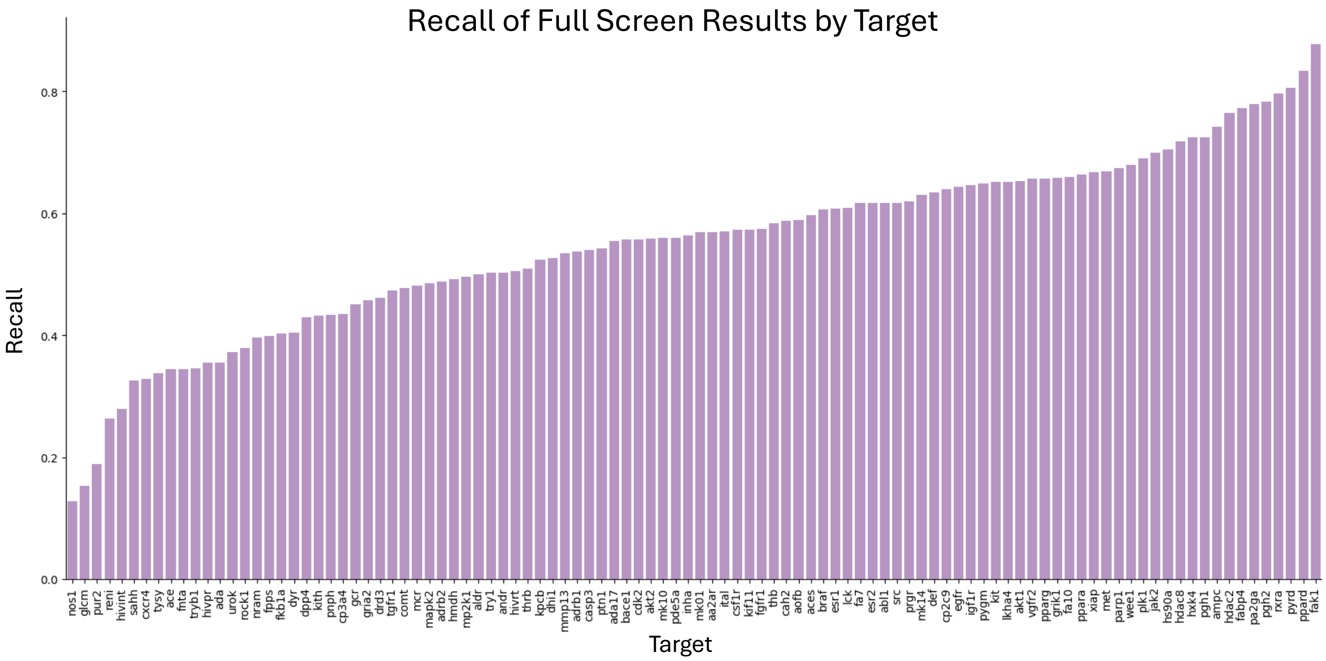

Figure S9. Recall values for full results across all queries for a target. Results were combined and then duplicates were removed.

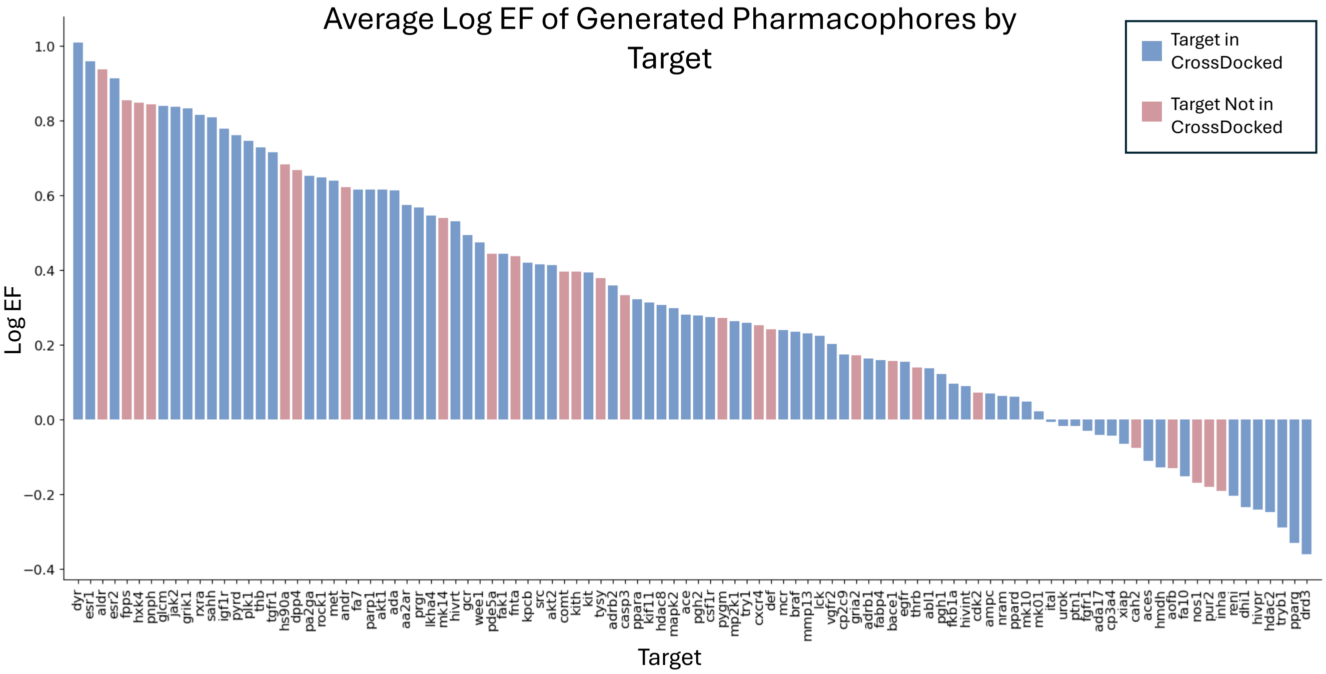

Figure S10. Comparison of average EF for pharmacophores generated for targets in CrossDocked versus those not in CrossDocked.

5 ADDITIONAL MINIMIZATION AND DOCKING RESULTS

We perform docking of ligands identified in CHEMBL by pharmacophores and ligands generated by *de novo* generative models using GNINA (Francoeur et al., 2020). We use the fast CNN setting of GNINA and report three metrics provided directly by GNINA: Vina affinity, CNN affinity, and CNN VS score (McNutt et al., 2021; Sunseri and Koes, 2021).

Figures S11 and S12 report the distribution of CNN affinity and CNN VS scores for ligands identified from virtual screening CHEMBL with generated pharmacophores and ligands generated with DiffSBDD and Pocket2Mol when minimized with their corresponding receptors. The top 100 ligands for each target are selected based on Vina affinity score.

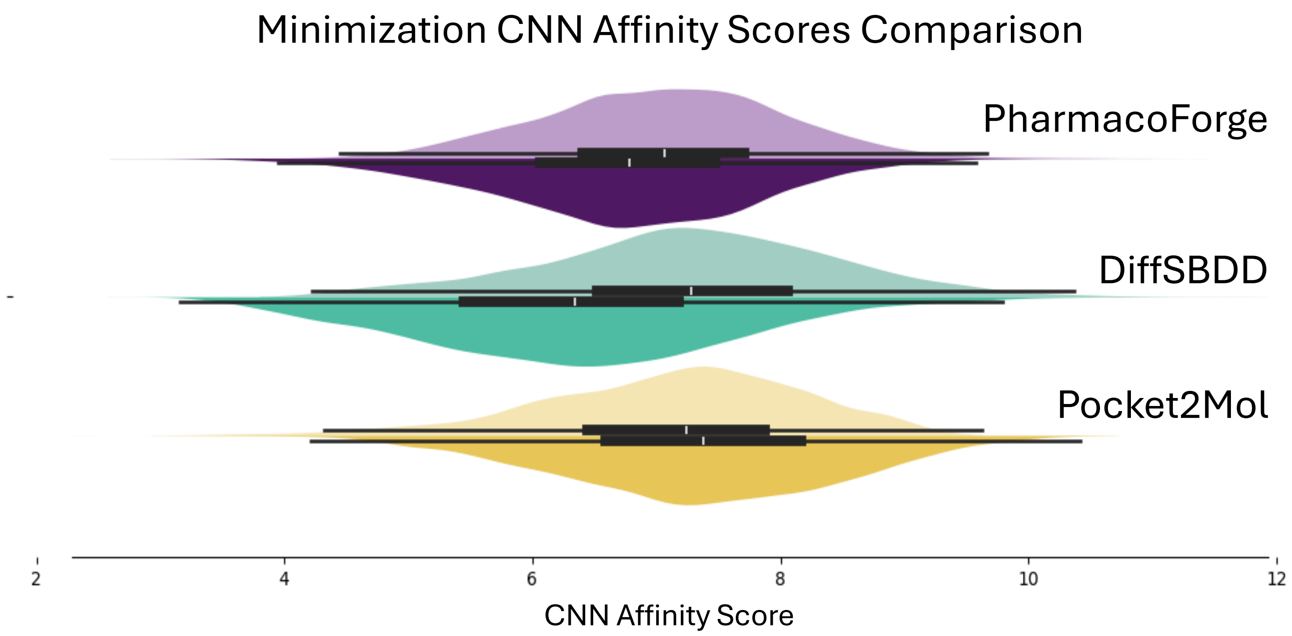

Figure S11. Distribution of CNN affinity scores from minimization. Results shown for the top 100 ligands for each DUD-E target. Original pose CNN affinity scores on top half of each violin plot with de-strained scores on the bottom half.

Figures S13 and S14 report the distribution of CNN affinity and CNN VS scores for ligands identified from virtual screening CHEMBL with generated pharmacophores and ligands generated with DiffSBDD and Pocket2Mol when docked with their corresponding receptors.

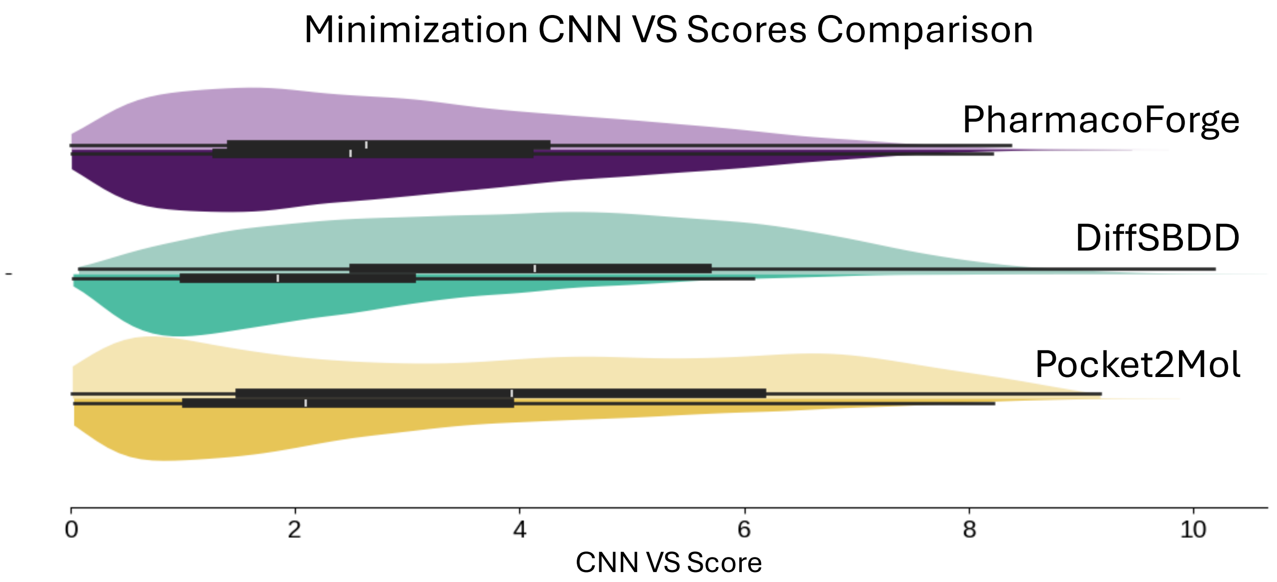

Figure S12. Distribution of CNN VS scores from minimization. Results shown for the top 100 ligands for each DUD-E target. Original pose CNN VS scores on top half of each violin plot with de-strained scores on the bottom half.

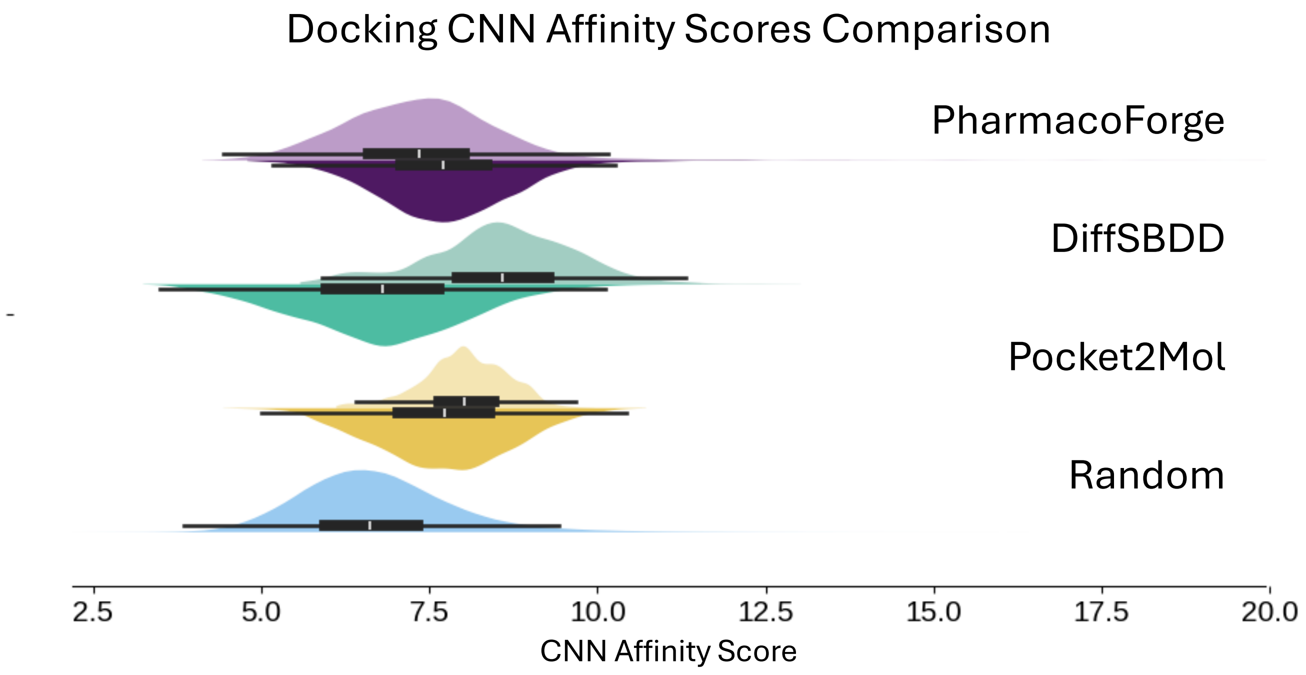

Figure S13. Distribution of CNN affinity scores from docking. Results shown for the top 100 ligands for each DUD-E target. Original pose CNN affinity scores on top half of each violin plot with de-strained scores on the bottom half. Scores are constrained to a maximum of 20 for plot visibility.

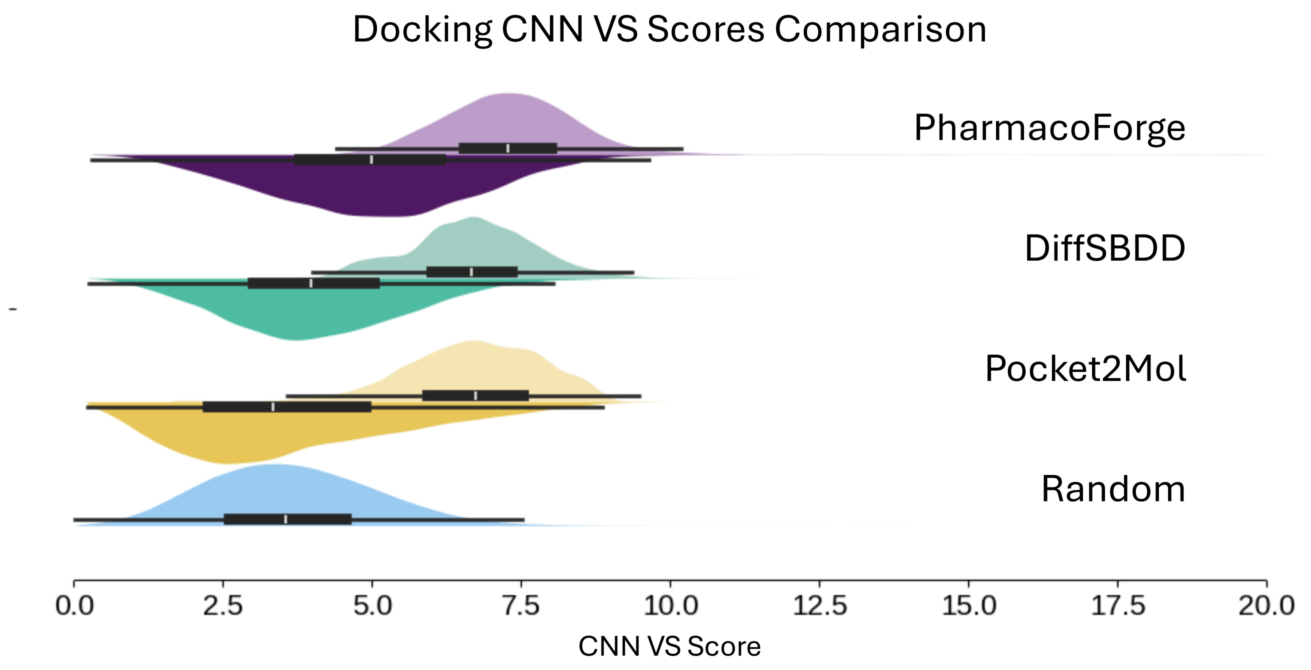

Figure S14. Distribution of CNN VS scores from docking. Results shown for the top 100 ligands for each DUD-E target. Original pose CNN VS scores on top half of each violin plot with de-strained scores on the bottom half. Scores are constrained to a maximum of 20 for plot visibility.

6 PHARMACOPHORE DISTRIBUTION AND EXAMPLES

6.1 PharmacoForge generates features at similar frequencies to the dataset

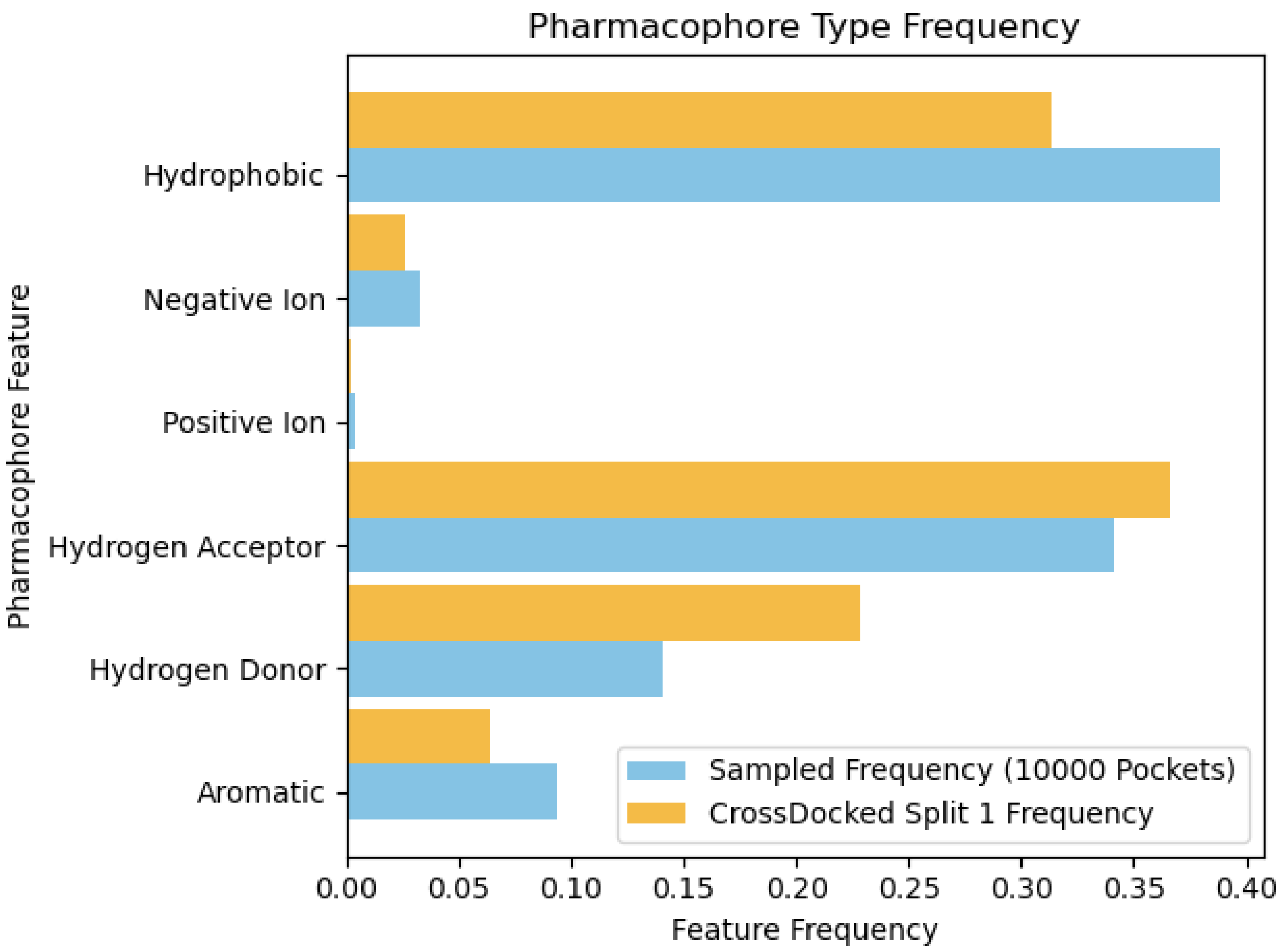

Figure S15. Frequency at which certain feature types are generated, comparing training data and pharmacophores generated by the model.

Figure S15 reports the frequency of occurrence of a pharmacophore feature type across the protein-ligand complexes present in the training set and compares it to the frequency from samples generated from the model (5 samples per pocket). The figure indicates that the model approximates the data distribution well as is evidenced by the agreement between the generated and train sets.

7 GENERATED PHARMACOPHORE EXAMPLES

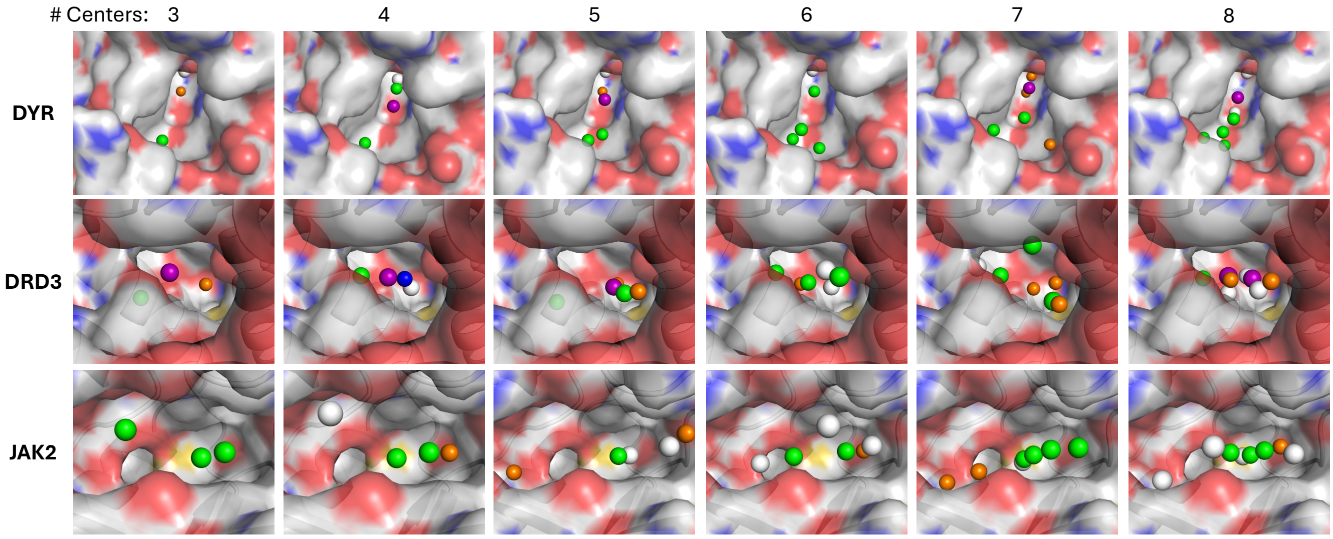

Figure S16. Generated pharmacophore samples for targets dihydrofolate reductase (DYR) (PDB:3NXO), dopamine D3 receptor (DRD3) (PDB: 3PBL), and tyrosine-protein kinase JAK2 (PDB: 3LPB)

Francoeur, P. G., Masuda, T., Sunseri, J., Jia, A., Iovanisci, R. B., Snyder, I., et al. (2020). Three-dimensional convolutional neural networks and a cross-docked data set for structure-based drug design. *Journal of Chemical Information and Modeling* 60, 4200–4215. doi:10.1021/acs.jcim.0c00411

Gale, D. and Shapley, L. (1962). College admissions and the stability of marriage. *The American Mathematical Monthly* 69, 9–15. doi:10.1080/00029890.1962.11989827

McNutt, A. T., Francoeur, P., Aggarwal, R., Masuda, T., Meli, R., Ragoza, M., et al. (2021). GNINA 1.0: Molecular docking with deep learning. *Journal of Cheminformatics* 13, 43. doi:10.1186/s13321-021-00522-2

Sunseri, J. and Koes, D. R. (2016). Pharmit: Interactive exploration of chemical space. *Nucleic Acids Research* 44, W442–W448. doi:10.1093/nar/gkw287

Sunseri, J. and Koes, D. R. (2021). Virtual screening with GNINA 1.0. *Molecules* 26, 7369. doi:10.3390/molecules26237369
